# Supplementary material for: Feline Demodicosis Case Report—First Molecular Characterization of Demodex Mites in Romania
Source: Pathogens. 2021 Nov 12;10(11):1474. doi: 10.3390/pathogens10111474 (PMC8622192; doi:10.3390/pathogens10111474)

File: 260_P2_DEMODEX_F.ab1 Run Ended: 2020/3/11 2:3:44 Signal G:283 A:475 C:271 T:580

Sample: 260_P2_DEMODEX_F Lane: 69 Base spacing: 12.844023 825 bases in 9804 scans

10 20 30 40 50 60 70 80 90 100 110 120

TT TTCATTTGGACTTGTATGAGGGGATTAATGAAGATTTTTTTATGAATGTTTTAATTGGTGAATTTAATTTTTCCATGAAAAATTGGAATTATTTCTAAAGACGAGAAGACCCCGAAATCTTTAT

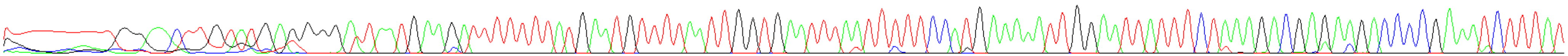

130 140 150 160 170 180 190 200 210 220 230 240 250

TTTAGAAAAAGGTATTTTAATTTTTATTTGGGGGAAAGTTTTATTTTTATATTTATTTTTATTATGAGGAACTTTATGAAGGTTGATTGGATAGATACTTCGGGGTTAACAGGATTATTTTTCTT

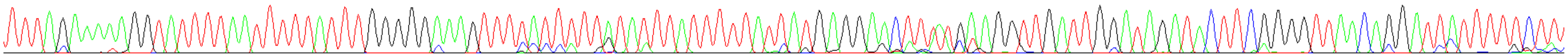

260 270 280 290 300 310 320 330 340 350 360 370

TGAAGTTCTTATTTTGGGGAGAGATTATTACCTCGATGTTGGCTTAAGAACGGACGGGCGTGGTCACAGGACAGCAACCCCACGCAATGTGCGTTGAGATTCATATTATATACGAAAATCTGATCG

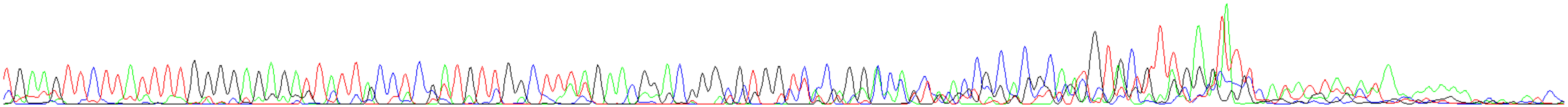

380 390 400 410 420 430 440 450 460 470 480 490 500

CTGCTACTGAGAAAGCCCTAGTCCGCGACAATATAGGCGTAGTGATGGTTTATATTCTGACTCACAGACTCGCGCGTCCTCAAGCTAAACCATCACGCAGTAGCGTTCAGAGGTAGGCGGCTAGGAGA

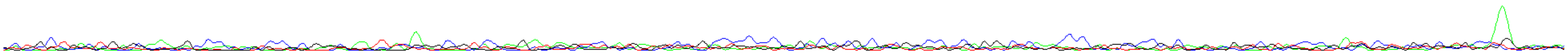

510 520 530 540 550 560 570 580 590 600 610 620 630

GAGAAGATCTGCATCATGTAGGCACTACAGTAGGCATATATGATGCGAGTGACAGTGATATGGTCATGATAGATTGCGGTACATTTGGTAGAGGGTGATGTTTAGACATGTCTACTTACGATATGAGAT

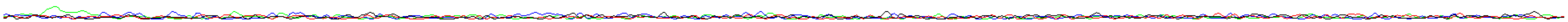

File: 260_P2_DEMODEX_F.ab1　*Run Ended: 2020/3/11 2:3:44*　*Signal G:283 A:475 C:271 T:580*

Sample: 260_P2_DEMODEX_F　*Lane: 69*　*Base spacing: 12.844023*　*825 bases in 9804 scans*　

640 650 660 670 680 690 700 710 720 730 740 750

TAAAATGTCGAATGCTATTTAATTGGCAGGAAAAGTTGGTTTATGGTCGTTAGCAGCGTAGATTAAGGATGAGATGATGGTATAGTGAGTAAGTAGTTGGGATTGTAGTTGGGGCGGTTGGTAT

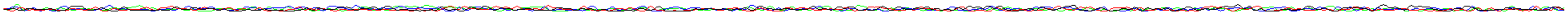

760 770 780 790 800 810 820

ATGCTGATATGCATAGGACTATAGGTTGCTGTAGTGGCTGTAGCTGAGAATTAATCGTTTAAAGATG

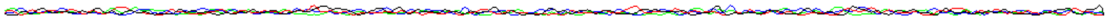

Supplement: Supplementary file 1 [file pathogens-10-01474-s001.zip › Supplementary Materials/S1_sequencing result_260_P2_DEMODEX_F.pdf]
