# Supplementary material for: Feline Demodicosis Case Report—First Molecular Characterization of Demodex Mites in Romania
Source: Pathogens. 2021 Nov 12;10(11):1474. doi: 10.3390/pathogens10111474 (PMC8622192; doi:10.3390/pathogens10111474)
Supplement: Supplementary file 1 [file pathogens-10-01474-s001.zip › Supplementary Materials/S2_sequencing result_260_P2_DEMODEX_R.pdf]

File: 260_P2_DEMODEX_R.ab1 Run Ended: 2020/3/11 2:3:44 Signal G:201 A:781 C:940 T:944

Sample: 260_P2_DEMODEX_R Lane: 67 Base spacing: 12.756867 756 bases in 9464 scans

10 20 30 40 50 60 70 80 90 100 110 120

GTAAGATAAGGAACTTCAAAGAGTATTATCCTGTTAACCCCGAAGTATCTATCCAATCAACCTACAAAAGTTCCTCATAATAAAAATAAATATAAAAATAAAACTTTCCCCCAAATAAAAATTAAA

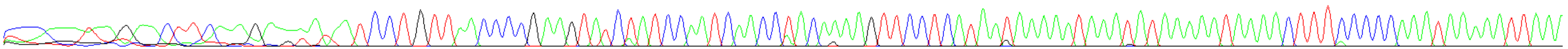

130 140 150 160 170 180 190 200 210 220 230 240

ATACCTTTTTCTAAAATAAAGATTTCGGGGTCTTCTCGTCTTTAGAAATAATTCCAATTTTTCATGGAAAAATTAAATTCACCAATTAAAACATTCATAAAAAAATCTTCATTAATCCCCTCA

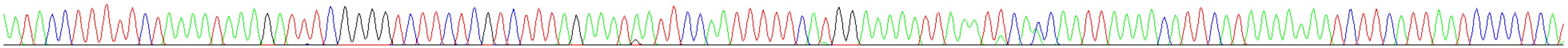

250 260 270 280 290 300 310 320 330 340 350 360 370

TACAAGTTTCCAATAAAAAAACAAATGACTTCGCTACCTTAGCACATACAATTAAGCGGGTGTGTTTTTGGTATTTGGTTGCGTTGTGGGGTGTTGGTTGTTGTTTTTGTTTGTGGGGTTGT

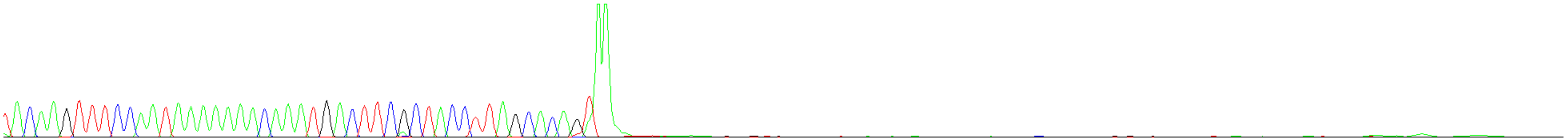

380 390 400 410 420 430 440 450 460 470 480 490

GTTTTTGTTTTTGGTGGGTTGGTTTGTTGGTTGGTTTGTGGTGTTGGTTTGGTTTGGTTGTGGGGGTGGGTTTGTTTGTTTGTGGTGGTTTTGGTGGGTTTTTTTTGGTTTTTTGGTTG

500 510 520 530 540 550 560 570 580 590 600

GTGGGGGTGTGTGTGTGGTGTGGTTTTTTTTTGGGTGTGGTTGGGGGGTTGGTGGGGTTTGGTTGTTGTGTTTTTGTGGTGGGTGTGTGGTGGGGTGTGTTGGTGGGGGGTGTTG

610 620 630 640 650 660 670 680 690 700 710 720

G T G G TT T G T GGG T G GGGTT G GTT T GGTTT GT GG G GGTTT G G T G T G T T G G G G T G T GG T G T GG G TT G T G TT GGG T T GT GT GG T T GG TT GGG G G T TGG G T T G GG GG GGGGT G T TTG G

---

730 740 750

TT T G T T GTGT GT TTT T T GT T T GG GGTTGG GT T G
